# Exploring mental health disparities in Mozambique: Depression and anxiety symptoms among reproductive-aged women using data from Mozambique Demographic and Health Survey 2022–23

**DOI:** 10.1016/j.gloepi.2025.100223

**Published:** 2025-09-30

**Authors:** Syed Toukir Ahmed Noor, Sazid Siddique, Oishi Das, Samin Yeasar, Raisha Binte Islam

**Affiliations:** a Department of Statistics, Shahjalal University of Science and Technology, Sylhet 3114, Bangladesh; b Department of Statistics, University of Dhaka, Dhaka 1000, Bangladesh.

**Keywords:** Anxiety, Depression, Mental health, Wealth-related inequality, Reproductive age, Mozambique

## Abstract

**Background:**

Mental health conditions, particularly symptoms of anxiety and depression among women of reproductive age, constitute a substantial public health burden. However, comprehensive studies on these issues are scarce in Mozambique.

**Objective:**

This study aims to investigate the prevalence and factors associated with depression and anxiety symptoms among Mozambican women of reproductive age using nationally representative data.

**Methods and materials:**

We analyzed data from the 2022–23 Mozambique Demographic and Health Survey, including a sample of 13,183 women aged 15–49. Depression and anxiety symptoms were assessed using the Patient Health Questionnaire (PHQ-9) and the Generalized Anxiety Disorder (GAD-7) scale. Multivariable logistic regression analysis was used to identify associated factors, and a concentration curve was employed to assess wealth-related inequality of mental health conditions.

**Results:**

Depression symptoms were reported by 10 % (95 % CI: 9.5–10.7) of women, while 11 % (95 % CI: 10.5–11.7) reported anxiety symptoms. Older age, skilled professions, and pregnancy were associated with higher odds of depression and anxiety symptoms. Conversely, women from wealthier households who engaged in agricultural work and had greater household decision-making power showed lower odds. Geographically, women in Nampula province had significantly higher odds, whereas those in Gaza province had lower odds. Also, significant wealth-related inequality was observed, with lower socioeconomic groups having higher mental health conditions.

**Conclusion:**

These findings highlight the urgent need for targeted interventions addressing socioeconomic and geographic disparities in mental health among Mozambican women. Efforts should focus on improving access to mental health services and integrating mental health care into broader public health strategies.

## Introduction

Mental health is defined as a condition of comprehensive well-being that includes cognitive, emotional, and social dimensions [1]. It influences how we manage stress, relate to others, and make life decisions [2]. Furthermore, among the several issues related to mental health, depression and anxiety are the most prevalent diseases globally [3,4]. Anxiety is characterized by the presence of worry, dread, or fear related to future events or situations. Depression, on the other hand, is a mood disorder that looks like constant feelings of sadness, hopelessness, and lack of concern for interesting activities [5,6]. Moreover, mental health has been recognized as a vital component of the Sustainable Development Goals (SDGs), specifically under Goal 3, which aims to ensure healthy lives and promote well-being for all at all ages [7].

About one-eighth of the world's population is affected by mental illness. Anxiety disorders are estimated to occur in 4 % of the global population, while depression affects 5 % [3]. Furthermore, there has been a rising trend in mental health issues among women of the reproductive age [8,9]. A study done by the Global Burden of Disease (GBD) in 2019 showed that the prevalence of mental disorders rose from 654.8 million to 970.1 million, a 48.1 % increase over the period between 1990 and 2019. However, this increase was slightly higher among females, at 50 % [10]. The same study also found that depression and anxiety were among the most disabling mental health conditions, ranking in the top quarter of the GBD list [10]. Research on mental health, particularly depression and anxiety, among women of reproductive age (15–49 years) is crucial for addressing their well-being [11].

According to previous studies, women in America were more likely to experience depression when living in state with high income inequality [12]. Similarly, in Spain, the probability of being diagnosed with anxiety or depression was 2.5-fold higher in women than in males [13]. Furthermore, the prevalence of depression and anxiety in Nigeria was 28.9 % each, with 19.8 % of individuals experiencing both conditions simultaneously [14]. In Indonesia, depression among young and middle-aged women in major cities was 15 %, and higher among lower socio-economic groups [15]. According to earlier research conducted in Nepal found that about 30 % of Nepalese people struggle with mental health issues [16], with the prevalence of depression and anxiety symptoms being 4 % and 17.7 %, respectively [17]. This study also reported that females were more likely to experience depression and anxiety compared to males [17]. Furthermore, in South Korea, 10.3 % of women of reproductive age were found to be psychologically unhealthy [18].

According to the World Health Organization (WHO), 4.1 % of the population had depressive disorders, and 3 % of the population had anxiety disorders in Mozambique [19]. Also, according to recent projections from the GBD study estimated that by 2050, depressive disorders will be the 9th leading cause of disease burden in Mozambique, with anxiety disorders ranked 19th [20]. In central Mozambique, a significant number of individuals experience depressive symptoms and lifetime suicidal thoughts. Despite the prevalence of these mental health issues, the majority go untreated, with 68 % of those with depressive symptoms and 89 % of those with suicidal ideation not receiving care [21]. Consequently, it was evident that rural Mozambique needs better access to and provision of mental health services [22,23]. In an ordinary hospital in Mozambique, there were 203 beds available for psychiatric patients in 2014, while the two psychiatric hospitals had 298 beds available [24]. Nevertheless, there is still a shortage of accessibility to mental health care despite the Ministry of Health's efforts, with just ten psychiatrists, 109 psychologists, and 23 occupational therapists in 2014 [24]. Additionally, a multi-country survey found that participants in Mozambique reported a lower psychological well-being than those in neighboring countries (Lesotho, Malawi, South Africa, Swaziland, Zambia, and Zimbabwe) [25].

This study addresses a critical knowledge gap by focusing specifically on the mental health burden among women of reproductive age in Mozambique, a population segment that is often underrepresented in national mental health planning and policy discourse [26]. Reproductive-aged women (15–49 years) face unique vulnerabilities due to biological, social, and economic factors, including gender-based violence, perinatal stress, limited autonomy in reproductive decision-making, and societal expectations surrounding motherhood [27,28]. These stressors place them at heightened risk for depression, anxiety, and suicidal ideation [27]. Additionally, poor mental health among women in this age group has direct intergenerational consequences, including adverse maternal and child health outcomes such as low birth weight, preterm birth, and impaired cognitive development in children [27]. Despite their central role in family and community health, reproductive-aged women are often overlooked in mental health research and service delivery in Mozambique [29]. Prioritizing this group is essential to achieving broader public health goals and advancing gender equity in mental health care access and outcomes [30].

Therefore, the study aims to estimate the burden of depression and anxiety symptoms among reproductive-aged women in Mozambique and investigate the associated factors and potential wealth-related disparities among women aged 15 to 49 years. To the best of our knowledge, this is the first study in Mozambique to report the prevalence and associated factors of depression and anxiety symptoms using data from a nationally representative survey, as the Mozambique Demographic and Health Survey (MDHS) 2022–23, which introduced a mental health module for the first time in its DHS survey history. Additionally, it investigates wealth-related inequities in a novel way, offering crucial baseline data to guide mental health interventions and policies for women of reproductive age. These findings will strengthen the current understanding and provide the groundwork for future studies aimed at developing and implementing targeted interventions to address mental health conditions among reproductive-aged women.

## Methodology

### Data source and sampling technique

For this research, we used data from the nationally representative cross-sectional MDHS 2022–2023, which was carried out between July 2022 and March 2023. The MDHS data is publicly available and can be accessed upon request from The DHS Program (link: https://www.dhsprogram.com/methodology/survey/survey-display-564.cfm).

The 2022–2023 Mozambique Demographic and Health Survey (MDHS) utilized a stratified, two-stage sampling design to provide estimates representative at the national level, as well as for urban and rural areas and the 10 provinces of Mozambique, including Maputo City, which holds provincial status. The sample frame was provided by the National Institute of Statistics (INE), based on the 2017 IV General Population and Housing Census (RGPH). In the first stage, 619 enumeration areas (EAs) were selected, with 232 EAs allocated to urban areas and 387 to rural areas. The selection of EAs was conducted using probability proportional to size (PPS), where the number of households in each stratum determined the probability of selection. Due to security concerns, eight districts in Cabo Delgado Province were excluded from the sampling frame. In the second stage, 26 households were systematically selected from each EA, resulting in a total of 16,045 households chosen for the survey. However, the final sample was slightly smaller than the target of 16,094 households because two selected EAs, one in Cabo Delgado and one in Zambézia could not be surveyed due to security issues. Of the 16,045 households selected, 14,250 were successfully interviewed. More details of the sampling can be found in the final report of the survey [31].

### Population and sample size

In this study, the target population comprised all reproductive-aged women (15–49 years) who participated in the survey. Within each selected household, all eligible women aged 15–49 years residing or staying in the household the prior night were approached for a face-to-face interview using a standardized women's questionnaire. Out of the 14,250 households successfully interviewed, 13,976 women aged 15 to 49 were identified as eligible for individual interviews. Of these, interviews were successfully completed with 13,183 women, resulting in a 94 % response rate. Finally, the study included 13,183 (weighted) women of reproductive age who responded to the mental health module of the survey. No other household members outside this age-sex eligibility window were asked the mental health module; thus, the study population is comprehensively representative of reproductive-aged women at the national level. The data were extracted from the women's individual record (IR) file of the standard DHS dataset for Mozambique.

### Study variables and measurements

#### Outcome variables

Women aged between 15 and 49 years were assessed for depression symptoms by the Patient Health Questionnaire (PHQ-9). It is a nine-item self-report tool that evaluates the presence of depression symptoms during the previous two weeks, with a total score that ranges from 0 to 27; the symptoms are classified as not depressive (0–4), mild (5–9), moderate (10–14), moderately severe (15–19), and severe (20–27). According to this study, depression symptoms were classified as present if a participant's score was 10 or above (coded as 1), and absent from the study if their score was less than 10 (coded as 0) [32]. Our study's PHQ-9 scale showed strong internal consistency, with a Cronbach's Alpha of 0.864.

The Generalized Anxiety Disorder Scale (GAD-7) was used to measure anxiety symptoms. Another self-reported scale consists of seven items that evaluate the occurrence and severity of generalized anxiety symptoms during the previous two weeks. Anxiety symptoms are recorded as missing (coded as 0) for scores below 10 and present (coded as 1) for values of 10 or higher, with the total score ranging from 0 to 21 [33]. A strong reliability was also shown by the GAD-7 scale, which has a Cronbach's Alpha coefficient of 0.857. Supplementary Table S1 contains the frequency distributions for the PHQ-9 and GAD-7 items.

#### Exploratory variables

This study incorporated a range of explanatory variables in such a way that they can capture socio-economic and demographic factors, maternal and paternal characteristics, household attributes, child-related factors, and regional characteristics of the respondents.

In case of socio-economic and demographic factors, variables included the age of the age of women (categorized as 15–19, 20–24, 25–29, 30–34, 35–39, 40–44, 45–49 years), their education level, and that of their husbands/partners (categorized as no education, primary, secondary, higher secondary or above). Additionally, the occupation of women was considered, including categories such as housewife, agriculture, professional/technical/managerial and clerical, skilled manual, and others. The age of husbands'/partners' (categorized as 15–19, 20–24, 25–29, 30–34, 35–39, 40–44, 45–49, 50+ years) and their occupation (jobless, agriculture, professional/technical/managerial and clerical, skilled manual, others) were also included. Marital status (never married, ever married), current pregnancy status (yes, no), mass media exposure (yes, no), and the number of major decisions of the household made by respondents on their own health care visit to the relative house and large household purchases (categorized as 0, 1, 2, 3) were further analyzed. For household attributes, the wealth index (categorized as poorest, poorer, middle, richer, richest) and number of household members (less or equal to four, four, or more) were considered. And lastly, regional-level variables included provinces and areas of residence, with residences classified as urban or rural.

### Statistical analysis

For this study, data cleaning, recoding, and analysis were conducted using Stata version 17.0 (StataCorp LP, College Station, Texas) following the DHS guidelines. Sample weights were used, and modifications were made to the intricate survey design, considering the primary sampling units (PSUs) and strata to guarantee the survey's representativeness [34]. The intricate survey design was managed with the help of the Stata “Svyset” tool. Moreover, the analysis followed STROBE standards for a cross-sectional study [35].

Descriptive statistics were employed to summarize the characteristics of the study variables. These included the reporting of frequencies and percentages for categorical variables. Then, chi-squared tests were performed to investigate the association between the categories and the outcome variables (depression and anxiety). Following that, a multiple logistic regression analysis was carried out to determine the characteristics that are linked with depression and anxiety symptoms in women aged 15 and 49 years. For the multivariable model, variables with a *P*-value of less than 0.20 in any bivariate analysis were considered for inclusion [36]. This study evaluated multicollinearity through the variance inflation factor (VIF), with the wealth index recording the highest VIF at 2.49 and an average VIF of 1.59 across all variables. The adjusted odds ratios (AOR) with confidence intervals (CI) of 95 % were used to present the strength of the relations.

### Wealth-related inequality

The study examined the relationship between wealth disparities and mental health conditions in women of reproductive age [37]. This was done by analyzing a concentration curve, which visually represented the distribution of depression and anxiety symptoms across different wealth groups. A positive Concentration Index (CIX) signifies a larger occurrence of mental health conditions among wealthy individuals. In contrast, a negative CIX indicates a higher burden of mental health conditions among individuals with lower socioeconomic levels. The CIX, which measures the level of socioeconomic inequality, was computed using the “convenient covariance” method [38]. A value of 0 on the CIX indicates an equal distribution across socioeconomic groups. The Stata command “lorenz” [38] and “conindex” [39] were employed to calculate the concentration curve and CIX, respectively.

## Results

Table 1 shows the distribution of socio-demographics and other characteristics among 13,183 reproductive-aged women (15–49 years). Among them, most participants were under 30 years old (23.1 % aged 15–19, 20.4 % aged 20–24, and 16.7 % aged 25–29), while over two-fifths of women had primary education (42.5 %). Furthermore, a large portion of respondents were housewives (65.6 %), and 31.3 % of their husbands were unemployed. Nearly one-third of the households were in the poor wealth quintile (36.3 %), and 1 in 2 respondents were not involved in any major household decisions (50.8 %). The respondents were predominantly from rural areas (61.2 %), with the highest proportion being from Nampula province (23.2 %).

**Table 1:** Descriptive statistics of study variables among Mozambican reproductive-aged women (*n* = 13,183).

Variables	Total
	Weighted frequency (%)
**Overall**	**13,183 (100)**
**Womens' Age**	
15–19	3050 (23.1)
20–24	2693 (20.4)
25–29	2195 (16.7)
30–34	1577 (12)
35–39	1486 (11.3)
40–44	1171 (8.9)
45–49	1011 (7.7)

**Womens' Education**	
No education	3522 (26.7)
Primary	5601 (42.5)
Secondary	3709 (28.1)
Higher secondary or above	352 (2.7)

**Husband/partners' Age**	
15–19	130 (1.5)
20–24	1084 (12.8)
25–29	1509 (17.8)
30–34	1414 (16.7)
35–39	1245 (14.7)
40–44	1114 (13.1)
45–49	892 (10.5)
50+	1098 (12.9)

**Husband/partners' Education**	
No education	1814 (25.1)
Primary	2984 (41.3)
Secondary	2141 (29.7)
Higher secondary or above	280 (3.9)

**Womens' Occupation**	
Housewife	8615 (65.6)
Agriculture	1780 (13.6)
Professional/Technical/ Managerial and Clerical	456 (3.5)
Skilled manual	1693 (12.9)
Others	584 (4.5)

**Husband/partners' Occupation**	
Jobless	2535 (31.3)
Agriculture	1580 (19.5)
Professional/Technical/ Managerial and Clerical	609 (7.5)
Skilled manual	2684 (33.2)
Others	685 (8.5)

**Marital status**	
Never married	2896 (22)
Ever married	10,287 (78)

**Wealth Index**	
Poorest	2420 (18.4)
Poorer	2363 (17.9)
Middle	2372 (18)
Richer	2810 (21.3)
Richest	3218 (24.4)

**No. of Household Members**	
≥4	4530 (34.4)
4>	8653 (65.6)

**Household Heads' Sex**	
Male	9001 (68.3)
Female	4182 (31.7)

**Mass Media Exposure**	
No	6687 (50.7)
Yes	6496 (49.3)

**No. of child (ever born)**	
0	3065 (23.3)
1	2131 (16.2)
2	2094 (15.9)
3	1803 (13.7)
4+	4089 (31)

**Number of major decisions of the household made by respondents**	
0	6703 (50.8)
1	833 (6.3)
2	1304 (9.9)
3	4344 (32.9)

**Currently Pregnant**	
No	12,211 (92.6)
Yes	972 (7.4)

**Area of residence**	
Urban	5120 (38.8)
Rural	8063 (61.2)

**Provinces**	
Maputo city	655 (5)
Niassa	861 (6.5)
Cabo Delgado	705 (5.3)
Nampula	3064 (23.2)
Zambézia	2193 (16.6)
Tete	1314 (10)
Manica	909 (6.9)
Sofala	909 (6.9)
Inhambane	555 (4.2)
Gaza	670 (5.1)
Maputo	1347 (10.2)

**Fig. 1:**
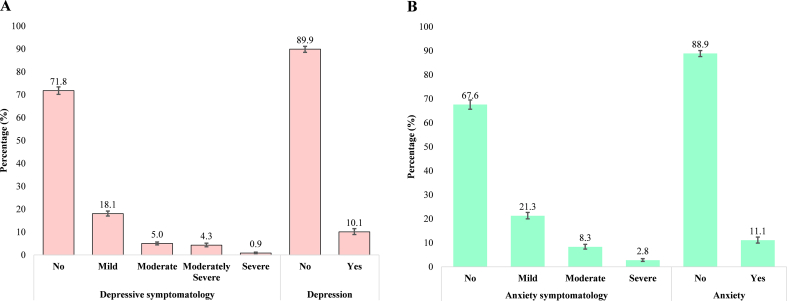
Prevalence and distribution of (a) depression and (b) anxiety symptoms among Mozambican reproductive-aged women (n = 13,183).

Fig. 1 illustrates the distribution of severity of depression and anxiety symptoms among reproductive-aged women at different levels. For depression, 71.8 % reported having no symptoms, 18.1 % showed mild symptoms, 5 % showed moderate symptoms, 4.3 % showed moderately severe symptoms, and 0.9 % showed severe symptoms. For anxiety, it was observed that 67.6 % of the individuals had no symptoms, 21.3 % had mild symptoms, 8.3 % had moderate symptoms, and 2.8 % had severe symptoms. We considered moderate to severe symptoms as the outcome. As a result, 10.1 % of respondents reported symptoms of depression, 11.1 % reported anxiety symptoms, 13.9 % experienced either anxiety or depression, and 7.3 % experienced both conditions concurrently.

Table 2 demonstrates the distribution of explanatory variables according to the experience of depression and anxiety symptoms among reproductive-aged women. Both depression and anxiety symptoms increased with respondents' age, peaking in women aged 45–49 (depression: 11.4 %, anxiety: 12.5 %). Women with only primary level education reported a higher prevalence of mental health conditions, with 11.5 % reporting depression and 12.1 % reporting anxiety symptoms. In the case of occupation status, the highest prevalence of depression (12.1 %) and anxiety symptoms (12.9 %) was reported by women who were housewives. Furthermore, women whose husbands were unemployed showed the highest burden of facing mental health conditions (depression: 15.6 %, anxiety: 16.9 %). Women who were married had a higher burden of mental health conditions, with 10.6 % of them reporting having depression and 12 % having anxiety symptoms. Furthermore, respondents from the poorest wealth quintile reported a higher prevalence of depression (15.8 %) and anxiety (16.7 %) compared to women from wealthier households. Respondents who were not exposed to mass media reported a higher prevalence of experiencing mental health conditions (depression:11.5 %, anxiety: 12.3 %), and pregnant women showed a high prevalence of mental health conditions, with 13.7 % reporting depression and 14.3 % reporting anxiety symptoms. Regarding geographical variations, the highest prevalence of mental health conditions was evident among women residing in Nampula provinces, while the lowest was among women living in Gaza.

**Table 2:** Distribution of study variables by depression and anxiety symptoms among Mozambican reproductive-aged women (*n* = 13,183).

Variables	Depression		Anxiety	
	n (row %)	*p-value*	n (row %)	*p-value*
**Overall**	**1337 (10.1)**		**1462 (11.1)**	
**Womens' Age**				
15–19	264 (8.6)	0.006	257 (8.4)	<0.001
20–24	302 (11.2)		311 (11.5)	
25–29	241 (11)		271 (12.4)	
30–34	153 (9.7)		176 (11.2)	
35–39	167 (11.3)		212 (14.3)	
40–44	95 (8.1)		108 (9.3)	
45–49	115 (11.4)		127 (12.5)	

**Womens' Education**				
No education	398 (11.3)	<0.001	418 (11.9)	0.005
Primary	645 (11.5)		679 (12.1)	
Secondary	276 (7.4)		342 (9.2)	
Higher secondary or above	18 (5.1)		23 (6.5)	

**Husband/partners' Age**				
15–19	16 (12.3)	0.915	13 (9.9)	0.995
20–24	102 (9.4)		114 (10.5)	
25–29	166 (11)		172 (11.4)	
30–34	158 (11.2)		166 (11.7)	
35–39	115 (9.3)		143 (11.5)	
40–44	113 (10.2)		127 (11.4)	
45–49	90 (10.1)		103 (11.6)	
50+	107 (9.7)		134 (12.2)	

**Husband/partners' Education**				
No education	191 (10.5)	0.012	196 (10.8)	0.370
Primary	271 (9.1)		304 (10.2)	
Secondary	168 (7.9)		198 (9.3)	
Higher secondary or above	7 (2.5)		19 (6.6)	

Womens**' Occupation**				
Housewife	1045 (12.1)	<0.001	1110 (12.9)	<0.001
Agriculture	80 (4.5)		81 (4.5)	
Professional/Technical/ Managerial and Clerical	15 (3.4)		32 (7)	
Skilled manual	159 (9.4)		196 (11.6)	
Others	32 (5.5)		41 (7)	

**Husband/partner**s**' Occupation**				
Jobless	397 (15.6)	<0.001	429 (16.9)	<0.001
Agriculture	86 (5.4)		101 (6.4)	
Professional/Technical/ Managerial and Clerical	38 (6.3)		58 (9.5)	
Skilled manual	252 (9.4)		273 (10.2)	
Others	70 (10.2)		79 (11.5)	

**Marital status**				
Never married	244 (8.4)	<0.047	229 (7.9)	<0.001
Ever married	1093 (10.6)		1233 (12)	

**Wealth Index**				
Poorest	383 (15.8)	<0.001	404 (16.7)	<0.001
Poorer	255 (10.8)		261 (11)	
Middle	206 (8.7)		229 (9.7)	
Richer	301 (10.7)		330 (11.7)	
Richest	192 (6)		238 (7.4)	

**No. of Household Members**				
≥4	474 (10.5)	0.558	529 (11.7)	0.297
4>	863 (10)		933 (10.8)	

**Household Heads' Sex**				
Male	912 (10.1)	0.954	1032 (11.5)	0.171
Female	425 (10.2)		431 (10.3)	

**Mass Media Exposure**				
No	769 (11.5)	0.003	824 (12.3)	0.007
Yes	568 (8.7)		638 (9.8)	

**No. of child (ever born)**				
0	270 (8.8)	0.303	276 (9)	0.154
1	239 (11.2)		263 (12.3)	
2	214 (10.2)		230 (11)	
3	202 (11.2)		239 (13.2)	
4+	411 (10.1)		455 (11.1)	

**Number of major decisions of the household made by respondents**				
0	805 (12)	<0.001	825 (12.3)	0.050
1	65 (7.8)		74 (8.9)	
2	100 (7.7)		127 (9.7)	
3	368 (8.5)		436 (10)	

**Currently Pregnant**				
No	1204 (9.9)	0.006	1323 (10.8)	0.019
Yes	133 (13.7)		139 (14.3)	

**Area of residence**				
Urban	489 (9.5)	0.473	554 (10.8)	0.749
Rural	848 (10.5)		908 (11.3)	

**Provinces**				
Maputo city	15 (2.3)		21 (3.2)	
Niassa	26 (3)	<0.001	24 (2.8)	<0.001
Cabo Delgado	92 (13)		64 (9.1)	
Nampula	742 (24.2)		873 (28.5)	
Zambézia	218 (9.9)		200 (9.1)	
Tete	38 (2.9)		68 (5.1)	
Manica	21 (2.3)		23 (2.5)	
Sofala	92 (10.1)		66 (7.2)	
Inhambane	18 (3.2)		9 (1.6)	
Gaza	6 (0.9)		7 (1.1)	
Maputo	70 (5.2)		108 (8)	

The associated factors of mental health conditions among Mozambican women of reproductive age are presented in Table 3. Women between the ages of 35 and 39 had a considerably higher risk of suffering both depression (AOR = 1.69, 95 % CI = 1.31, 2.18) and anxiety (AOR = 2.09, 95 % CI = 1.63, 2.66) symptoms compared to women aged 19 or younger. Women who completed primary school showed a 16 % higher likelihood of experiencing depression symptoms (AOR = 1.16, 95 % CI = 1.00, 1.35) and a 20 % higher likelihood of anxiety symptoms (AOR = 1.20, 95 % CI = 1.03, 1.39). Respondents who worked in skilled professions had a higher chance of experiencing depression (AOR = 1.26, 95 % CI = 1.02, 1.54) and anxiety (AOR = 1.49, 95 % CI = 1.23, 1.81). Ever-married women experienced a 35 % higher likelihood of anxiety symptoms (AOR = 1.35, 95 % CI = 1.09, 1.67) compared to never-married women. Women from the richest household had a considerably lower likelihood of depression (AOR = 0.61, 95 % CI = 0.45, 0.82) and anxiety symptoms (AOR = 0.59, 95 % CI = 0.44, 0.78). Respondents who made more major household decisions had lower odds of both depression and anxiety, and pregnant women had a significantly higher risk of experiencing both depression (AOR = 1.35, 95 % CI = 1.09, 1.66) and anxiety (AOR = 1.27, 95 % CI = 1.04, 1.57) symptoms. Regarding provinces, respondents living in Nampula had significantly greater chances of experiencing depression (AOR = 11.09, 95 % CI = 6.40, 19.22) and anxiety symptoms (AOR = 11.89, 95 % CI = 7.37, 19.19) compared to women in Maputo City. On the other hand, women in Gaza had a far lower likelihood of experiencing depression (AOR = 0.34, 95 % CI = 0.13, 0.89) and anxiety symptoms (AOR = 0.29, 95 % CI = 0.12, 0.69).

**Table 3:** Associated factors of depression and anxiety among Mozambican reproductive-aged women (*n* = 13,183).

	Depression		Anxiety	
Variables	AOR (95 % CI)	*p-value*	AOR (95 % CI)	*p-value*
**Womens' Age**				
15–19	Ref.		Ref.	
20–24	1.37 (1.12, 1.68)	0.002	1.35 (1.1, 1.66)	0.004
25–29	1.34 (1.07, 1.67)	0.011	1.42 (1.14, 1.77)	0.002
30–34	1.32 (1.03, 1.7)	0.03	1.43 (1.12, 1.83)	0.005
35–39	1.69 (1.31, 2.18)	<0.001	2.09 (1.63, 2.66)	<0.001
40–44	1.21 (0.9, 1.62)	0.201	1.3 (0.98, 1.73)	0.067
45–49	1.66 (1.26, 2.2)	<0.001	1.72 (1.3, 2.26)	<0.001

**Womens' Education**				
No education	Ref.		Ref.	
Primary	1.16 (1, 1.35)	0.044	1.2 (1.03, 1.39)	0.016
Secondary	1.09 (0.88, 1.35)	0.449	1.42 (1.15, 1.74)	0.001
Higher secondary or above	1.38 (0.78, 2.43)	0.268	1.38 (0.81, 2.34)	0.233

Women**s' Occupation**				
Housewife	Ref.		Ref.	
Agriculture	0.6 (0.46, 0.78)	<0.001	0.64 (0.49, 0.83)	0.001
Professional/Technical/ Managerial and Clerical	0.49 (0.28, 0.87)	0.014	0.94 (0.61, 1.44)	0.777
Skilled manual	1.26 (1.02, 1.54)	0.029	1.49 (1.23, 1.81)	<0.001
Others	0.97 (0.66, 1.44)	0.895	1.14 (0.79, 1.63)	0.482

**Marital status**				
Never married	Ref.		Ref.	
Ever married	1.15 (0.93, 1.42)	0.187	1.35 (1.09, 1.67)	0.005

**Wealth Index**				
Poorest	Ref.		Ref.	
Poorer	0.72 (0.6, 0.86)	<0.001	0.71 (0.59, 0.85)	<0.001
Middle	0.72 (0.59, 0.88)	0.001	0.78 (0.64, 0.94)	0.01
Richer	0.84 (0.68, 1.04)	0.104	0.83 (0.68, 1.03)	0.085
Richest	0.61 (0.45, 0.82)	0.001	0.59 (0.44, 0.78)	<0.001

**Mass Media Exposure**				
No	Ref.		Ref.	
Yes	1.15 (0.98, 1.33)	0.078	1.14 (0.98, 1.32)	0.09

**Number of major decisions of the household made by respondent**s				
0	Ref.		Ref.	
1	0.66 (0.5, 0.87)	0.003	0.8 (0.61, 1.04)	0.096
2	0.58 (0.46, 0.73)	<0.001	0.75 (0.6, 0.93)	0.01
3	0.6 (0.52, 0.69)	<0.001	0.64 (0.55, 0.74)	<0.001

**Currently Pregnant**				
No	Ref.		Ref.	
Yes	1.35 (1.09, 1.66)	0.005	1.27 (1.04, 1.57)	0.022

**Provinces**				
Maputo city	Ref.		Ref.	
Niassa	1.21 (0.62, 2.36)	0.579	0.9 (0.48, 1.67)	0.728
Cabo Delgado	5.68 (3.17, 10.19)	<0.001	3.05 (1.79, 5.22)	<0.001
Nampula	11.09 (6.4, 19.22)	<0.001	11.89 (7.37, 19.19)	<0.001
Zambézia	3.97 (2.26, 6.97)	<0.001	3.11 (1.89, 5.1)	<0.001
Tete	1.41 (0.75, 2.64)	0.282	2.01 (1.19, 3.4)	0.01
Manica	0.77 (0.39, 1.54)	0.46	0.65 (0.35, 1.21)	0.176
Sofala	5 (2.83, 8.84)	<0.001	2.59 (1.54, 4.37)	<0.001
Inhambane	1.28 (0.63, 2.6)	0.5	0.46 (0.2, 1.02)	0.057
Gaza	0.34 (0.13, 0.89)	0.027	0.29 (0.12, 0.69)	0.005
Maputo	2.13 (1.21, 3.74)	0.009	2.42 (1.5, 3.92)	<0.001

**Fig. 2:**
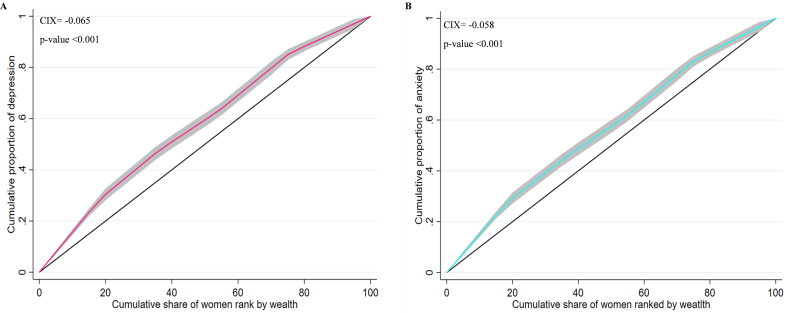
Concentration curve for depression (A) and anxiety (B) against wealth index among Mozambican reproductive-aged women (*n* = 13,183). *CIX: Concentration Index*

Fig. 2 shows the difference in mental health conditions across different wealth groups among reproductive-aged women in Mozambique. The concentration curve showed a significant wealth-related inequality between depression and anxiety since it consistently appears above the equality line. Furthermore, the CIX values provided additional evidence for this disparity, with CIX = −0.058 (*p*-value <0.001) for anxiety and CIX = −0.065 (p-value <0.001) for depression. These negative CIX values imply that women from lower socioeconomic backgrounds were more likely to have depression and anxiety symptoms.

## Discussion

In this study, the primary goal was to examine the mental health conditions of Mozambican women of reproductive age, focusing specifically on symptoms of depression and anxiety. According to our findings, 10 % of respondents reported symptoms of depression, and 11 % reported anxiety symptoms. These estimates are slightly higher than the 2021 GBD estimates, which reported a prevalence of anxiety disorders at 7.0 % (95 % CI: 4.8–10.0) and depressive disorders at 7.3 % (95 % CI: 5.7–9.6) among Mozambican women aged 15–49 years [40]. Our findings suggest that self-reported symptoms from a nationally representative community-based survey may reflect a more accurate and current epidemiological picture than statistical models alone. Furthermore, a previous study conducted in rural Mozambique found that 14 % of female heads of household had depressive symptoms [41]. According to another cross-sectional study conducted in Mozambique, the prevalence of anxiety was 13.3 %, and depressive symptom was 8.6 % [42]. However, it's important to note that neither of these estimates is nationally representative, limiting the generalizability of their findings to the wider Mozambican population.

Mental health symptom patterns were significantly associated with several sociodemographic and reproductive factors. We found that older women within the reproductive age group were more likely to experience symptoms of both depression and anxiety. According to an earlier primary study and a systematic review, older women are more likely to suffer from mental disease throughout midlife transitions and report higher levels of depression and anxiety symptoms [43,44]. Also, depression and anxiety symptoms in older women may increase due to biological factors, comorbidities, menopause, and changes in marital status, parenting duties, and perceived health as they age [45]. Within the Mozambican context, older reproductive-aged women are more likely to have larger families and greater domestic burdens, including caregiving for multiple children or grandchildren, especially in multigenerational and extended households [46]. These caregiving and household demands, often compounded by limited access to mental health care or social support systems, may contribute to higher psychological distress.

Furthermore, it was also evident that the occupation of reproductive-aged women was associated with depression and anxiety symptoms, as women who worked in agriculture had lower odds of depression and anxiety. A study found that farm residence is associated with better mental health outcomes compared to isolated rural residence for women of reproductive age [47]. Additionally, another study found that farming habit was associated with a lower risk of depressive symptoms, partly due to increased physical activity [48]. On the other hand, we also found that skilled manual laborers had a higher risk of depression and anxiety among women of reproductive age, similar to previous findings showing that working mothers experience higher levels of these conditions compared to non-working mothers [49]. High levels of work stress, such as an overwhelming workload and time constraints, could be the reason behind it [50]. However, a study conducted in India found that homemakers had higher anxiety and stress levels compared to working women [51].

In this study, a significant association was found between marital status and mental health conditions, with ever-married women more likely to have these symptoms than never-married women. Some previous studies also found that married women were at higher risk for depression and anxiety due to factors such as reduced personal control, increased role demands, and relationship issues [52]. However, a few studies presented opposite findings, suggesting that marital status may not always increase mental health risks [53,54]. These variations in findings may reflect differences in psychosocial aspects of marital roles, caregiving demands, and the availability of social support systems within marriages [55,56]. In the context of Mozambique, cultural expectations and gender norms may exacerbate emotional distress among married women, particularly due to societal pressure to conform to traditional roles, early marriage, and economic dependence on spouses [42]. Additionally, the potential lack of emotional support from partners and exposure to gender-based violence, often underreported in surveys, may further contribute to mental health vulnerability [57]. These contextual factors suggest that the quality and dynamics of marital relationships, rather than marital status alone, are critical determinants of women's mental well-being.

Our study also found that women's decision-making autonomy within households plays a significant protective role against depression and anxiety. Having greater control over household decisions was associated with higher self-esteem, confidence, and improved coping mechanisms, which together help buffer psychological distress [58]. This observation is supported by prior research demonstrating an inverse relationship between women's involvement in household decision-making and the prevalence of anxiety and depressive symptoms [59,60]. In Mozambique, where traditional patriarchal norms frequently restrict women's participation in key family decisions, these findings are especially relevant [61]. Recent nationally representative data indicate that Mozambican women who are actively involved in decisions about their own healthcare, large household purchases, and family visits report notably fewer mental health symptoms compared to those with limited or no decision-making power [60]. These results highlight that efforts to enhance women's autonomy, particularly in settings characterized by entrenched gender roles, can serve as an effective approach not only to advance gender equity but also to improve mental health outcomes across communities.

The present study also found that currently pregnant women had a significantly higher risk of depression and anxiety, possibly due to pregnancy risk and gestational age influencing stress and access to resources [62]. Additionally, hormonal changes during pregnancy, particularly fluctuations in estrogen, progesterone, and cortisol, may adversely affect mental health and increase the likelihood of anxiety disorders [63,64]. Symptoms such as nausea, vomiting, and fatigue in early pregnancy have also been linked to depressive symptoms [65]. Similar findings have been reported in previous studies, which identified pregnancy as a risk factor for depression [66,67].

This study identified significant disparities in depression and anxiety levels among women of reproductive age across different provinces of Mozambique. These differences can be attributed to a combination of socio-economic, environmental, and health-related factors that vary across different provinces. In particular, women residing in provinces such as Nampula, Cabo Delgado, Sofala, and Zambézia had notably higher odds of experiencing both depression and anxiety symptoms as compared to those living in Maputo City. A previous study also found that depression and anxiety were common among internally displaced individuals affected by armed violence in Cabo Delgado, Mozambique, particularly among females [68]. Additionally, women in Sofala, exhibited a higher likelihood of seeking medical attention for neurotic or stress-related ailments [69]. Depression affects 14 % of female heads of households in rural Mozambique, especially Zambézia, and risk factors include distance from clinics and food shortages [41]. On the other hand, we found that women in Gaza had a lower risk of developing depression and anxiety than in Maputo city. This phenomenon may be attributed to the presence of robust community support structures, cultural values that foster resilience, or enhanced availability of resources in contrast to the more isolated northern provinces.

Lastly, our study also revealed significant inequality in the mental health outcomes of Mozambican women, particularly in relation to wealth disparities. The concentration curve analysis consistently fell below the line of equality for both depression and anxiety, indicating that women with lower socioeconomic status faced a greater burden of mental health conditions. The finding aligns with several previous studies, which found that poverty or lower income or lower socioeconomic status is associated with higher risks of depression and anxiety in women of reproductive age [70,71]. It could be because of economic stress, limited access to healthcare, or inadequate social support [70,72]. Also, it was observed in studies conducted across different contexts, including Uganda [73], the USA [12,74], and New Zealand [75], where financial hardship has been consistently linked to increased risks of depression and anxiety.

Together, these findings link the mental health of reproductive-aged women not only to biological and demographic variables but to deeper structural inequalities related to gender roles, economic position, and health system limitations. The high mental health burden identified in certain provinces, such as Cabo Delgado and Zambézia, highlights the need for decentralized and context-specific mental health services in Mozambique. Improving maternal mental health outcomes in Mozambique will require multisectoral action, including: 1) integrating mental health screening into existing maternal and child health services; 2) deploying task-sharing with community health workers to address human resource shortages; and 3) prioritizing gender-sensitive policies that address social norms and economic vulnerabilities. Addressing the psychological well-being of reproductive-aged women is not only essential for individual health but also central to improving family and community health outcomes more broadly.

## Strengths and limitations

One of the major strengths of this study is the inclusion of a large, nationally representative sample of 13,183 Mozambican women, which improves its statistical power and generalizability. Additionally, the study's detailed focus on both depression and anxiety symptoms offers a broad understanding of mental health issues among women of reproductive age in Mozambique. A key contribution is the use of concentration curve analysis to evaluate wealth-related inequalities in mental health, providing valuable insights into the socioeconomic disparities that affect mental health outcomes. Another major strength is the use of DHS data, where data collection is usually conducted by highly trained local personnel, ensuring data reliability and accuracy. Also, depression and anxiety symptoms were measured using widely recognized and validated tools, such as the GAD and PHQ scales, adding further credibility to the findings.

Along with its strengths, the study has certain drawbacks that should be considered. First, the cross-sectional design limits the ability to establish causal linkages between variables, as data were collected at a single point in time. Second, the assessment of depression and anxiety symptoms relied on self-reported responses to screening tools, which introduces the potential for recall bias, underreporting, or social desirability bias, particularly in stigmatized contexts. Third, while the study includes a range of sociodemographic and household-level variables available in the DHS dataset, it lacks key psychosocial and behavioral variables, such as perceived social support, community participation, access to mental health services, history of gender-based violence, substance use, physical comorbidities, and medication use, which are known to impact mental health. The absence of such variables limited our ability to assess a broader range of contributing factors. This limitation is primarily due to the nature of secondary data; the DHS methodology does not collect detailed psychosocial or clinical information relevant to mental health beyond standard demographic and reproductive health indicators. Future studies, particularly those involving primary data collection, should incorporate these critical variables to enable more comprehensive and contextually grounded analyses. Finally, while validated screening tools (PHQ-9 and GAD-7) were used to capture symptoms, they do not constitute clinical diagnoses. Future research could benefit from incorporating clinical diagnostic interviews and longitudinal designs to more properly explore the complex pathways linking sociocultural factors and mental health outcomes.

## Conclusion

This study provides nationally representative insights into the prevalence and determinants of depression and anxiety symptoms of Mozambican women of reproductive age. Key factors associated with mental health conditions were age, education, occupation, marital status, wealth index, higher major making decisions, and current pregnancy status. The protective role of women's autonomy highlights the need for policies that promote female empowerment and gender equity in household and community decision-making. The heightened risk among low-income groups and certain provinces underscores the urgency of expanding equitable access to mental health services, particularly through integrating screening and support within maternal and primary healthcare systems. Addressing occupational stress, providing targeted psychosocial support during pregnancy, and tailoring interventions for vulnerable regions can help mitigate the mental health burden. Further research and comprehensive approaches are needed to improve mental health services and reduce inequalities, contributing to better mental health and well-being among women in Mozambique.

## CRediT authorship contribution statement

**Syed Toukir Ahmed Noor:** Writing – original draft, Visualization, Project administration, Methodology, Formal analysis, Data curation, Conceptualization. **Sazid Siddique:** Writing – original draft, Visualization, Resources, Methodology, Data curation. **Oishi Das:** Writing – original draft, Software, Resources, Data curation. **Samin Yeasar:** Writing – original draft, Software, Resources, Data curation. **Raisha Binte Islam:** Writing – review & editing, Validation, Supervision, Investigation.

## Consent for publication

Not applicable.

## Ethics approval and consent to participate

We do not need ethical approval as we used the secondary data from DHS. However, details of ethical approval for DHS are available at: https://dhsprogram.com/Methodology/Protecting-the-Privacy-of-DHS-Survey-Respondents.cfm. The survey was approved by the Ethics Committee of the ICF International at Rockville, Maryland, USA, and by the Ministry of Health and Family Welfare Ethics Committee. The study is conducted using the principles of the Declaration of Helsinki. All NDHS participants provided written informed consent before participation, and all information was collected confidentially.

## Declaration of generative AI and AI-assisted technologies in the writing process

During the preparation of this work, the author(s) used Grammarly, QuillBot, and ChatGPT 4o to enhance the manuscript's language quality. After using these tools, the author(s) reviewed and edited the content as needed and take(s) full responsibility for the content of the publication.

## Funding

The authors did not receive any specific grant from funding agencies in the public, commercial, or not-for-profit sectors.

## Declaration of competing interest

There are no potential conflicts (financial, professional, or personal) for any of the authors to disclose.

## Data Availability

Data are available on request from the DHS program website (The DHS Program - Mozambique: Standard DHS, 2022-23 Dataset).

## Appendix A Supplementary data

Supplementary material Image 1
